# Chinese Version of the Mobile Health App Usability Questionnaire: Translation, Adaptation, and Validation Study

**DOI:** 10.2196/37933

**Published:** 2022-07-06

**Authors:** Yi Shan, Meng Ji, Wenxiu Xie, Rongying Li, Xiaobo Qian, Xiaomin Zhang, Tianyong Hao

**Affiliations:** 1 School of Foreign Studies Nantong University Nantong China; 2 School of Languages and Cultures University of Sydney Sydney Australia; 3 Department of Computer Science City University of Hong Kong Hong Kong China; 4 School of Artificial Intelligence South China Normal University Guangzhou China; 5 School of Computer Science South China Normal University Guangzhou China; 6 Department of Linguistics Macquarie University Sydney Australia

**Keywords:** mHealth app, usability, Chinese version of MAUQ, improved translation, validity, stability, reliability, cross-cultural adaptability, mobile phone

## Abstract

**Background:**

The usability of mobile health (mHealth) apps needs to be effectively evaluated before they are officially approved to be used to deliver health interventions. To this end, the mHealth App Usability Questionnaire (MAUQ) has been designed and proved valid and reliable in assessing the usability of mHealth apps. However, this English questionnaire needs to be translated into other languages, adapted, and validated before being utilized to evaluate the usability of mHealth apps.

**Objective:**

This study aims to improve, further adapt, and validate the Chinese version of the MAUQ (C-MAUQ; interactive for patients) on *Left-handed Doctor*, one of the most popular “reaching out to patients” interactive mHealth apps with chatbot function in China, to test the reliability and cross-cultural adaptability of the questionnaire.

**Methods:**

The MAUQ (interactive for patients) has been translated into Chinese and validated for its reliability on *Good Doctor*, one of the most influential “reaching out to patients” mHealth apps without chatbot function in China. After asking for the researchers’ approval to use this Chinese version, we adjusted and further adapted the C-MAUQ by checking it against the original English version and improving its comprehensibility, readability, idiomaticity, and cross-cultural adaptability. Following a trial survey completed by 50 respondents on *wenjuanxing*, the most popular online questionnaire platform in China, the improved version of the C-MAUQ (I-C-MAUQ) was finally used to evaluate the usability of *Left-handed Doctor* through an online questionnaire survey (answered by 322 participants) on *wenjuanxing*, to test its internal consistency, reliability, and validity.

**Results:**

The I-C-MAUQ still retained the 21 items and 3 dimensions of the original MAUQ: 8 items for usability and satisfaction, 6 items for system information arrangement, and 7 items for efficiency. The translation problems in the C-MAUQ, including (1) redundancy, (2) incompleteness, (3) misuse of parts of speech, (4) choice of inappropriate words, (5) incomprehensibility, and (6) cultural difference–induced improper translation, were improved. As shown in the analysis of data obtained through the online survey, the I-C-MAUQ had a better internal consistency (ie, the correlation coefficient between the score of each item and the total score of the questionnaire determined within the range of 0.861-0.938; *P*<.01), reliability (Cronbach α=.988), and validity (Kaiser–Meyer–Olkin=0.973), compared with the C-MAUQ. It was effectively used to test the usability of *Left-handed Doctor*, eliciting over 80% of informants’ positive attitudes toward this mHealth app.

**Conclusions:**

The I-C-MAUQ is highly reliable and valid for *Left-handed Doctor*, and suitable for testing the usability of interactive mHealth apps used by patients in China. This finding further confirms the cross-cultural validity, reliability, and adaptability of the MAUQ. We identified certain factors influencing the perceived usability of mHealth apps, including users’ age, gender, education, profession, and possibly previous experience with mHealth apps and the chatbot function of such apps. Most notably, we found a wider acceptance of this new technology among young Chinese female college students who were more engaged in the interaction with health care chatbots. The age-, gender-, and profession-induced preference for new digital health interventions in China aligns with the findings in other similar studies in America and Malaysia. This preference identifies areas for further research on the social, cultural, and gender adaptation of health technologies.

## Introduction

### Background

Mobile health (mHealth) apps have been applied to deliver health interventions (eg, health education, health monitoring, recommendations on treatments) to alleviate the overburdened health systems in many countries. These apps can perform versatile tasks, including health management, behavior intervention, health data collection, self-diagnosis, disease management, medication management, rehabilitation, and acting as patient portals [1,2], improving medication compliance, saving time in diagnosis and treatment, and reducing medical costs [3-6]. Given these wide applications and diverse advantages, these apps need to be assessed for hidden expenses, heavy data entry burden, and interest loss [7] to ensure accurate data analysis before being put into use [8].

To effectively evaluate the usability of mHealth apps, different questionnaires were designed [9], among which the most popular are the System Usability Scale (SUS) and the Post-Study System Usability Questionnaire (PSSUQ) [10,11]. Although used to reliably measure certain usability aspects of mobile apps, the SUS and the PSSUQ, among others, failed to provide tailored information on the factors unique to mobile apps [10,12]. Zhou et al [9] developed and validated the mHealth App Usability Questionnaire (MAUQ), which was solely designed for assessing the usability of mHealth apps, attesting its reliability and validity. The MAUQ [9] was exclusively developed to evaluate the usability of mHealth apps. It has 4 versions designed to assess interactive or standalone mHealth apps among patients or health care providers. It shows a strong internal consistency, evidenced by the Cronbach α coefficients of its 3 dimensions (.895 for *ease of use and satisfaction*, .829 for *system information arrangement*, and .900 for *usefulness*) and the overall Cronbach α of .914. The items in the 3 dimensions are rated on a 7-point Likert scale from 1 (extremely strongly agree) to 7 (extremely strongly disagree). The usability of an app can be determined by calculating the total points and determining the average points of the responses to all statements: the closer the average is to 1, the higher the usability of the app [9].

Two more recent studies translated and adapted the MAUQ into Chinese [13] and Malay [14], respectively, finding that the Chinese and Malay versions exhibited high reliability and validity similar to those of the original English version [13,14]. The Chinese version of the MAUQ (C-MAUQ; interactive for patients) was testified to be reliable and valid, with content validity index of 0.952, Cronbach α of .912, value of test-retest reliability of 0.896, and value of the split-half reliability of 0.701 [14]. The Malay version of the MAUQ (standalone for patients) was proved to be reliable for evaluating the usability of the mHealth apps (Cronbach α=.946) [13]. Considering the painstaking efforts and considerable time and cost investment involved in developing new questionnaires [14], Marzuki et al [12] strongly recommended that established, accessible, and reliable questionnaires should be adapted, validated, and recorded cross-linguistically.

*Left-handed Doctor* is one of the most popular “reaching out to patients” [15] interactive mHealth apps in China. It integrates artificial intelligence technologies, such as deep learning, big data processing, semantic understanding, and interactive medical dialog with medicine and is committed to using artificial intelligence technology to expand the supply of high-quality medical resources. The *Left-handed Doctor* open platform provides solutions, such as smart hospitals, diagnostic robots for consultation rooms, intelligent online consultation, intelligent postdiagnosis management, and artificial intelligence internet hospitals. In combination with different application scenarios, it provides high-quality medical services for all parties, empowering the health care industry. Although it is popular among many people in China, no studies have empirically tested its usability using the C-MAUQ.

### Objective

Informed by the MAUQ and its culturally adapted versions, this study aimed to testify further the reliability, validity, and cross-cultural adaptability of the MAUQ for its suitability to the mHealth app usability test. This was achieved by applying the improved version of the C-MAUQ (I-C-MAUQ) to *Left-handed Doctor*, one of the most popular “reaching out to patients” interactive mHealth apps with chatbot function in China. Two facts warrant this study: (1) the *Left-handed Doctor* app is different from the *Good Doctor* app: the former is empowered with the chatbot function, while the latter is not, and we thought that this difference would influence users’ perceived usability of these apps; and (2) the informants differ from those in Mustafa et al [13] in terms of age, gender, education, and profession, and we believed that these differences would also impact users’ perceived usability of these apps.

## Methods

### Overview

This study used the C-MAUQ [15] but made some improvements. The study was conducted from February 18 to March 8, 2022.

### Improvement of the C-MAUQ

We first obtained the approval of the researchers [15] to use the C-MAUQ. Afterward, 2 translators (YS and MJ) independently adjusted this version by checking it against the original English version and improving its readability and idiomaticity. The I-C-MAUQ still retained the 21 items and 3 dimensions of the original MAUQ and the C-MAUQ: 8 items for usability and satisfaction, 6 items for system information arrangement, and 7 items for efficiency. Improper translations of all the 21 items in the C-MAUQ were modified through discussion among the whole research team.

### Improvement of Cross-cultural Adaptation

The C-MAUQ has been adapted cross-culturally through experts’ comments and a prediction test [15]. Based on this adaptation and drawing on Conway et al’s translatability assessment (TA) [16], this study further adapted the C-MAUQ by inviting a group of bilingual translators and health educators to assess the comprehensibility of the content as well as the cultural relevance and appropriateness of each item. Subsequently, the revised version was subjected to a trial survey online, in which 50 college students participated to identify problems that needed to be resolved.

### Informants and Online Survey

Participants were students of the School of Foreign Studies, Nantong University, China. Impacted by varying degrees of psychological problems that became increasingly serious during the repeated COVID-19 attacks, these students urgently needed mHealth apps for self-diagnosis and general health information to relieve their psychologically strained minds. The questionnaire was administered using the online questionnaire survey platform named *wenjuanxing* [17] on February 18, 2022, and the survey lasted until no additional questionnaire was submitted online for 2 consecutive days (March 4, 2022). Over this period, the survey was announced to the entire student body of over 1000 at the School of Foreign Studies, Nantong University, through emails and WeChat groups. Meanwhile, the candidate informants were requested to use the *Left-handed Doctor* app for 2 days to become familiar with it before answering the questionnaire. The majority of participants in this study were female, which is characteristic of all schools of foreign studies in China.

### Data Collection

The survey was conducted through *wenjuanxing* [17], the most popular online questionnaire platform in China. Two categories of data were collected via online questionnaires: the demographic information of the participants and their ratings on the 21 items concerning the usability of *Left-handed Doctor*. The demographic data included the informants’ age, gender, grade, and channel to obtain health care information. The usability test elicited data concerning the informants’ ratings of the 21 items based on a 7-point Likert scoring system from 1 to 7 points (representing “strongly agree,” “agree,” “somewhat agree,” “neither agree nor disagree,” “somewhat disagree,” “disagree,” and “strongly disagree,” respectively).

### Data Analysis

Quantitative analyses were conducted using SPSS version 22.0 (IBM, Inc.). First, demographic data were presented in a table and briefly described as the background information of the analysis. Subsequently, item analysis, weight analysis, and Pearson correlation analysis were conducted, followed by the reliability, validity, test-retest reliability, and split-half reliability tests. Finally, the range, mean values, and SD of the collected usability data were calculated and described for each of the 21 items.

### Ethics Considerations

This study was approved and supported by the Student Affairs Office and the Humanities and Social Sciences Office of Nantong University, which is authorized to provide such approval before collecting data from students.

## Results

### Improvement of the C-MAUQ

Both translators (YS and MJ) found items 1, 2, 5, 9, 11-14, 17-21 problematic after checking the C-MAUQ against the original English version independently. They modified these items independently, and then, through discussion, agreed on the corresponding revisions and the classification of translation problems, which were subjected to further amendments before a final consensus among the study researchers. The translation problems in the C-MAUQ were related to (1) redundancy (items 1, 2, and 18); (2) incompleteness (item 12); (3) misuse of parts of speech (items 5, 9, and 17); (4) choice of inappropriate words (items 5, 9, 14, and 18-21); and (5) incomprehensibility (items 9, 11, and 13).

### Further Cross-cultural Adaptation

The I-C-MAUQ was further adapted cross-culturally through a panel meeting attended by a group of bilingual translators and health educators. This meeting identified and agreed on a common problem concerning inappropriate cultural adaptation of items 18-21. In English-speaking countries, a patient always visits the same doctor and addresses the doctor as “my health care provider.” By contrast, in China, a patient usually sees different doctors when becoming ill and thus never uses “my” when referring to his/her “health care provider.” Therefore, “my” was crossed out from these 4 items. No other problems were detected during the panel meeting. After the panel meeting, the comprehensibility, readability, idiomaticity, and cultural adaptability of the questionnaire content were further improved. Subsequently, the I-C-MAUQ version was validated in an online trial survey completed by 50 informants. The trial survey turned out to be successful (Cronbach α=.992), and so the I-C-MAUQ did not require further improvement. The I-C-MAUQ, together with the C-MAUQ and the MAUQ, is provided in Multimedia Appendix 1.

### Informant Demographics

Multimedia Appendix 2 shows the informants’ demographic information. A total of 322 responses were collected online, including 292 (90.7%) from female respondents. This can be explained by the fact that over 90% of students studying in the School of Foreign Studies, Nantong University, are females. The age of the participants ranged from 18 to 33 years (mean 21.68, SD 2.30 years). The overwhelming majority (n=316, 98.1%) were aged between 18 and 26 years. The informants included freshman (n=64, 19.9%), sophomore (n=29, 9.0%), junior (n=88, 27.3%), senior (n=48, 14.9%), first-year postgraduate candidates (n=46, 14.3%), and second-year postgraduate candidates (n=47, 14.6%). The majority of the informants (n=306, 95.0%) obtained health care information by visiting a doctor; logging into the internet; and communicating with families, friends, and classmates. Only a minor percentage of participants (n=9, 2.8%) used mHealth apps to obtain health care information.

### Questionnaire Item Analysis

The 21 items in the I-C-MAUQ were valid and appropriately designed (Table 1), as evidenced by the distinction between the high-score group (n=94) and the low-score group (n=149). Data below the 27% quantile belonged to the low-score group, and those above the 73% quantile belonged to the high-score group. There was a significant difference in each of the 21 items between the high-score group and the low-score group, with *P* value in each case being <.001 (ie, *P*<.01). This indicates that all 21 items could well be distinguished from one another and thus should all be retained in the final version of the questionnaire. Besides, all the 21 items were significant (Table 2), with critical values (CR) determined within the range of 14.751-19.449 and the *P* value (CR) calculated at <.001 (ie, *P*<.01). The correlation coefficient between the score of each item and the total score of the questionnaire was determined within the range of 0.861-0.938 (*P*<.01). Thus, all the 21 items were retained. According to the Pearson correlation values (Table S1 of Multimedia Appendix 3), all the 21 items were significantly and positively correlated, with the correlation coefficients ranging from 0.688 to 0.921 and *P*<.01.

**Table 1:** Item analysis.

Items^a^	Group, mean (SD)			*t* (critical values)		*P* value^b^
	Low-score group (*n*=149)	High-score group (*n*=94)				
1	1.58 (0.57)	3.65 (1.08)	17.031		<.001	
2	1.52 (0.51)	3.34 (1.12)	14.751		<.001	
3	1.56 (0.55)	3.54 (1.02)	17.251		<.001	
4	1.58 (0.54)	3.66 (1.08)	17.352		<.001	
5	1.58 (0.58)	3.51 (1.08)	15.961		<.001	
6	1.57 (0.56)	3.53 (1.07)	16.348		<.001	
7	1.58 (0.55)	3.85 (1.05)	19.449		<.001	
8	1.56 (0.52)	3.62 (1.06)	17.555		<.001	
9	1.61 (0.61)	3.60 (1.17)	15.225		<.001	
10	1.53 (0.51)	3.61 (1.03)	18.186		<.001	
11	1.52 (0.51)	3.49 (1.05)	16.905		<.001	
12	1.58 (0.57)	3.46 (0.99)	16.724		<.001	
13	1.52 (0.54)	3.40 (1.04)	16.262		<.001	
14	1.56 (0.52)	3.65 (1.04)	18.038		<.001	
15	1.55 (0.53)	3.55 (1.06)	16.993		<.001	
16	1.56 (0.56)	3.56 (1.11)	16.178		<.001	
17	1.56 (0.52)	3.51 (1.09)	16.242		<.001	
18	1.55 (0.53)	3.54 (1.04)	17.182		<.001	
19	1.55 (0.55)	3.50 (1.03)	16.832		<.001	
20	1.67 (0.67)	3.68 (0.98)	17.527		<.001	
21	1.56 (0.52)	3.57 (1.08)	16.862		<.001	

^a^Items 1-21 represent the 21 items in the questionnaire.

^b^All *P* values <.01.

**Table 2:** Correlation between the 21 items and the overall score of the questionnaire.

Items	CR^a^	*P* value (CR)	COSQ^b^	*P* value^c^ (COSQ)
1	17.031	<.001	0.874	<.001
2	14.751	<.001	0.885	<.001
3	17.251	<.001	0.902	<.001
4	17.352	<.001	0.907	<.001
5	15.961	<.001	0.861	<.001
6	16.348	<.001	0.883	<.001
7	19.449	<.001	0.890	<.001
8	17.555	<.001	0.921	<.001
9	15.225	<.001	0.879	<.001
10	18.186	<.001	0.925	<.001
11	16.905	<.001	0.938	<.001
12	16.724	<.001	0.923	<.001
13	16.262	<.001	0.906	<.001
14	18.038	<.001	0.923	<.001
15	16.993	<.001	0.914	<.001
16	16.178	<.001	0.879	<.001
17	16.242	<.001	0.910	<.001
18	17.182	<.001	0.912	<.001
19	16.832	<.001	0.896	<.001
20	17.527	<.001	0.869	<.001
21	16.862	<.001	0.905	<.001

^a^CR: critical value.

^b^COSQ: correlation with the overall score of the questionnaire.

^c^All *P* values <.01.

### Weight of the 21 Items in the Questionnaire

Through the analytic hierarchy process, the weight of each of the 21 items in the questionnaire was determined. Based on the judgment matrix of the 21 items (Table S2 of Multimedia Appendix 3), the eigenvector and weight of each item were determined (Table 3). Drawing on the eigenvectors, the maximum eigenvalue (21.000) was worked out. According to the maximum eigenvalue, the CI (<0.001) was computed. According to Table 4, the random index (RI) of the judgment matrix was 1.6358. From the CI (<0.001) and the RI (1.6358), CR (<0.001) was finally calculated (Table 5). This CR value (<0.1) indicated that the judgment matrix passed the consistency test. Therefore, the weights of the 21 items in Table 3 were valid. These weight values meant that the 21 items were almost equally important in the questionnaire.

**Table 3:** Analytic hierarchy process analysis of the 21 items in the questionnaire^a^.

Items	Eigenvectors	Weight (%)
1	1.018	4.846
2	0.954	4.541
3	0.990	4.712
4	1.010	4.808
5	1.020	4.858
6	1.007	4.795
7	1.071	5.099
8	0.990	4.712
9	1.015	4.833
10	0.992	4.725
11	0.956	4.554
12	0.980	4.668
13	0.956	4.554
14	1.015	4.833
15	0.994	4.731
16	0.983	4.681
17	0.996	4.744
18	1.004	4.782
19	0.995	4.738
20	1.056	5.029
21	0.999	4.757

^a^Maximum eigenvalue: 21.000; CI<0.001.

**Table 4:** RI^a^ table of the judgment matrix.

Order	3	4	5	6	7	8	9	10	11	12	13	14	15	16
RI	0.52	0.89	1.12	1.26	1.36	1.41	1.46	1.49	1.52	1.54	1.56	1.58	1.59	1.5943
Order	17	18	19	20	21	22	23	24	25	26	27	28	29	30
RI	1.6064	1.6133	1.6207	1.6292	1.6358	1.6403	1.6462	1.6497	1.6556	1.6587	1.6631	1.6670	1.6693	1.6724

^a^RI: random index.

**Table 5:** Consistency test of the weight of the 21 items.

Maximum eigenvalue	CI	RI^a^	Critical value	Result of test
21.000	<0.001	1.636	<0.001	Pass

^a^RI: random index.

### Questionnaire Reliability and Validity

The statistics in Table 6 indicate the high reliability of the questionnaire. The corrected item-total correlation values of the 21 items all fell within 0.845-0.931, far exceeding 0.4. This meant that the 21 items were strongly correlated, and that they all had a high degree of reliability. Besides, the Cronbach α did not apparently increase when each of the 21 items was deleted, which implied that all items should be retained in the questionnaire. The overall Cronbach α (.988) for the 21 items was well above 0.9, indicating that the data collected for each item in the questionnaire were highly reliable. The values of test-retest reliability and split-half reliability were 0.918 and 0.828, respectively. Therefore, all the data were suitable for further analysis.

**Table 6:** Questionnaire reliability (and internal consistency).

Items	Corrected item-total correlation	Cronbach α if item deleted^a^
1	0.860	.988
2	0.873	.988
3	0.891	.988
4	0.897	.988
5	0.845	.988
6	0.870	.988
7	0.877	.988
8	0.912	.987
9	0.866	.988
10	0.917	.987
11	0.931	.987
12	0.915	.987
13	0.896	.988
14	0.914	.987
15	0.905	.987
16	0.866	.988
17	0.900	.987
18	0.902	.987
19	0.885	.988
20	0.855	.988
21	0.895	.988

^a^Cronbach α (standardized)=.988.

Table 7 reveals that the questionnaire is highly valid. The communalities for all 21 items ranged from 0.738 to 0.881, well above 0.4, indicating that the data can effectively be extracted from all these items. The Kaiser–Meyer–Olkin (KMO) value (0.973) was above 0.9, which showed that all the data concerning the 21 items could effectively be extracted. The percentage of variance (rotated) for factor 1 was 81.053%, considerably above 50%, meaning that all the data on all the items can validly be extracted.

**Table 7:** Questionnaire validity.

Items	Factor loadings (factor 1)	Communalities^a^
1	0.873^b^	0.762
2	0.885^b^	0.784
3	0.902^b^	0.813
4	0.907^b^	0.822
5	0.859^b^	0.738
6	0.882^b^	0.778
7	0.889^b^	0.790
8	0.921^b^	0.848
9	0.878^b^	0.771
10	0.925^b^	0.856
11	0.939^b^	0.881
12	0.924^b^	0.854
13	0.907^b^	0.823
14	0.923^b^	0.852
15	0.915^b^	0.837
16	0.880^b^	0.774
17	0.911^b^	0.830
18	0.912^b^	0.832
19	0.896^b^	0.803
20	0.868^b^	0.754
21	0.905^b^	0.819
Eigenvalues (initial)	17.021	N/A^c^
Variance (%) (initial)	81.053	N/A^c^
Cumulative variance (%) (initial)	81.053	N/A^c^
Eigenvalues (rotated)	17.021	N/A^c^
Variance (%) (rotated)	81.053	N/A^c^
Cumulative variance (%) (rotated)	81.053	N/A^c^
Kaiser–Meyer–Olkin	0.973	N/A^c^
Bartlett test of sphericity (chi-square); *df*	10873.765; 210	N/A^c^
*P* value	<.001	N/A^c^

^a^The communality is less than 0.4.

^b^The absolute value of loading is greater than 0.4.

^c^N/A: not applicable.

### Usability of the *Left-handed Doctor* App

Table 8 presents the results of the descriptive analysis of the usability of *Left-handed Doctor*. The range, mean (SD), and median scores were based on the rating of each item (1=strongly agree; 2=agree; 3=somewhat agree; 4=neither agree nor disagree; 5=somewhat disagree; 6=disagree; and 7=strongly disagree). The mean scores of the 21 items were between 2.224 and 2.497, indicating that the respondents were inclined to agree with the statements in all 21 items. In other words, they found the *Left-handed Doctor* app usable on the whole.

There were no significant differences (*P*=.35) in the mean scores concerning the 3 dimensions of usability and satisfaction (items 1-8), the arrangement of system information (items 9-14), and efficiency (items 15-21). This implied that the participants found the *Left-handed Doctor* app equally usable when it comes to the 3 dimensions.

Multimedia Appendix 4 shows the proportion of respondents falling into each of the 7 ratings of the 21 items. Over 60% (205/322, 63.7%; 223/322, 69.3%; 209/322, 64.9%; 206/322, 64.0%; 199/322, 61.8%; 206/322, 64.0%; 210/322, 65.2%; 203/322, 63.0%; 208/322, 64.6%; 219/322, 68.0%; 211/322, 65.5%; 218/322, 67.7%; 198/322, 61.5%; 208/322, 64.6%; 216/322, 67.1%; 207/322, 64.3%; 198/322, 61.5%; 203/322, 63.0%; 208/322, 64.6%, for items 1-6, 8-19, and 21, respectively) of informants *strongly agreed* or *agreed* with all items but items 7 (183/322, 56.8%) and 20 (187/322, 58.1%). More than 80% (267/322, 82.9%; 285/322, 88.5%; 277/322, 86.0%; 277/322, 86.0%; 271/322, 84.2%; 277/322, 86.0%; 259/322, 80.4%; 282/322, 87.6%; 270/322, 83.9%; 279/322, 86.6%; 287/322, 89.1%; 285/322, 88.5%; 288/322, 89.4%; 277/322, 86.0%; 280/322, 87.0%; 280/322, 87.0%; 282/322, 87.6%; 276/322, 85.7%; 276/322, 85.7%; 258/322, 80.1%; 277/322, 86.0%, for items 1-21, respectively) of participants *strongly agreed*, *agreed*, or *somewhat agreed* with all the 21 items. This meant that the vast majority of the participating students showed a positive attitude toward the usability of the *Left-handed Doctor* app.

**Table 8:** Descriptive analysis of the usability of the *Left-handed Doctor* app.

Item	Samples, n	Range	Mean (SD)	Median
1	322	1.000-7.000	2.373 (1.180)	2.000
2	322	1.000-7.000	2.224 (1.079)	2.000
3	322	1.000-7.000	2.307 (1.125)	2.000
4	322	1.000-7.000	2.354 (1.160)	2.000
5	322	1.000-7.000	2.379 (1.176)	2.000
6	322	1.000-7.000	2.348 (1.170)	2.000
7	322	1.000-7.000	2.497 (1.246)	2.000
8	322	1.000-7.000	2.307 (1.136)	2.000
9	322	1.000-7.000	2.366 (1.182)	2.000
10	322	1.000-7.000	2.314 (1.140)	2.000
11	322	1.000-7.000	2.230 (1.101)	2.000
12	322	1.000-7.000	2.286 (1.070)	2.000
13	322	1.000-7.000	2.230 (1.089)	2.000
14	322	1.000-7.000	2.366 (1.153)	2.000
15	322	1.000-7.000	2.317 (1.132)	2.000
16	322	1.000-7.000	2.292 (1.153)	2.000
17	322	1.000-7.000	2.323 (1.117)	2.000
18	322	1.000-7.000	2.342 (1.125)	2.000
19	322	1.000-7.000	2.320 (1.119)	2.000
20	322	1.000-7.000	2.463 (1.166)	2.000
21	322	1.000-7.000	2.329 (1.137)	2.000

## Discussion

### Principal Findings

Informed by Zhou et al [9] and Mustafa et al [13], the study improved the C-MAUQ translated, adapted, and validated in Zhao et al [14], and then used the I-C-MAUQ to test the usability of *Left-handed Doctor*, one of the most popular “reaching out to patients” interactive mHealth apps in China. The I-C-MAUQ had a better internal consistency (the correlation coefficient between the score of each item and the total score of the questionnaire ranging from 0.861 to 0.938; *P*<.001), reliability (Cronbach α=.988), validity (load factor ranging from 0.859 to 0.939, percentage of cumulative variance [rotated]=81.053%, KMO=0.973), test-retest reliability (0.918), and split-half reliability (0.828) than the C-MAUQ [14]. Such better performance of the I-C-MAUQ resulted from 4 factors: (1) better comprehensibility, readability, and cultural adaptation of the I-C-MAUQ; (2) different categories of participants in terms of age, gender, education, profession, and sample size; (3) different functions of the tested interactive mHealth apps used by patients (with vs without the chatbot function); and (4) respondents’ experience with mHealth apps. Similarly, we found that the reliability of the I-C-MAUQ was relatively higher than those reported in Mustafa et al [13] (Cronbach α=.946; corrected item-total correlation values between –0.057 and 0.868) and Zhou et al [9] (Cronbach α=.914). We once again attributed the reliability difference to the aforesaid 4 factors, which will be discussed in the following sections.

### Cross-cultural Adaptation of the Translated Questionnaire

It is imperative to adapt questionnaires cross-culturally, but there is a lack of evidence for the best approaches to cross-cultural adaptation (CCA) [18]. The most adopted methods for CCA are Brislin’s Translation Model [19], the use of panels or committees [20-26], and focus groups [27]. However, this study adopted another effective but a commonly neglected model: TA [16]. Drawing on the cross-cultural issues proposed in TA, we improved the C-MAUQ [15] by making further cultural and linguistic adaptations, solving the translation problems concerning redundancy, incompleteness, misuse of parts of speech, choice of inappropriate words, incomprehensibility, and relevance and appropriateness on the cultural, semantic, syntactic, and pragmatic facets. The newly adapted questionnaire was equivalent to the original questionnaire [18]. TA thus makes it possible to identify alternative versions for translation purposes, modify original versions to optimize subsequent translation efforts, and detect and discuss irrelevant or inappropriate items early [16]. Thus, TA needs to be adopted as an effective CCA method in prospective translation and adaptation of questionnaires.

### Participant Differences in Age, Gender, Education, Profession, and Sample Size

Most (318/322, 98.8%) of the informants in this study were aged 18-28, compared with the majority (91.04%) of respondents aged 29-65 in Zhao et al [14], with just over half (52.3%) of the participants aged 18-28 and just below half (48.3%) aged 29-65 in Zhou et al [9], and with all (100%) those surveyed aged 22-25 in Mustafa et al [13]. We concluded that younger age potentially led to relatively positive ratings of questionnaire items and thus higher questionnaire reliability and internal consistency.

The proportions of male and female participants (30/322, 9.3% vs 291/322, 90.4%) were different from those (53.76% vs 46.24%) in Zhao et al [14], those (38.3% vs 61.7%) in Zhou et al [9], and those (8% vs 92%) in Mustafa et al [13]. Therefore, considerably higher percentages (292/322, 90.7%) of female respondents seemed to contribute to a higher degree of the questionnaire’s internal consistency and reliability. This result showed that females were more interested in participating in surveys on the usability of mHealth apps and that more female users of mobile apps were keen on using mHealth apps for health care. This has been also testified by Zhou et al [9].

All informants in this study and Mustafa et al [13] were college students at the undergraduate or graduate level, but those in Zhao et al [14] and Zhou et al [9] had different levels of education: 33.24% and 67.2% held an undergraduate or above in Zhao et al [14] and Zhou et al [9], respectively. The overall higher level of respondent education may explain the relatively higher degree of questionnaire’s internal consistency and reliability in our study and Mustafa et al [13], in comparison with that in Zhao et al [14] and Zhou et al [9]. However, the vast gap in participant education at or above the undergraduate level between Zhao et al [14] and Zhou et al [9] merely resulted in a considerably minor difference in questionnaire reliability (Cronbach α=.912 vs .914).

In terms of profession, being a student—100% (322/322) in this study and Mustafa et al [13], 31.4% in Zhou et al [9], and 1.56% in Zhao et al [14]—also likely impacted the questionnaire’s internal consistency and reliability, with the rate of students participating positively proportional to the degree of reliability and internal consistency.

These findings concerning age, gender, education, and profession contradicted the result in Zhou et al [9], which asserted that the demographic factors (eg, age, gender, education, occupation) failed to significantly impact the answers to the individual statements or the overall score on the MAUQ.

The sample size was indeed not a contributing factor to the high internal consistency and reliability of the questionnaire. Zhao et al [14] recruited the largest number of participants (n=346) but reported the lowest internal consistency and reliability, whereas this study achieved the highest internal consistency and reliability of the questionnaire based on the data contributed by a similar number of informants (n=322), followed by a slightly lower internal consistency and reliability derived from the information provided by the smallest number of informants in Mustafa et al [13].

### Respondents’ Experience With mHealth Apps

The informants in Zhou et al [9] used mobile apps for an average of 6.64 years; 86.42% of participants in Zhao et al [14] used mHealth apps more than 3 times during the month before the survey. Only 2.8% (9/322) of respondents in this study resorted to mHealth apps for health care information, but they were requested to install the *Left-handed Doctor* app 2 weeks beforehand to become familiar with it. The informants in Mustafa et al [13] were also asked to do the same. Therefore, experience with mHealth apps did not seem to influence the users’ perceived usability, and thus the internal consistency and reliability of the questionnaire adopted remained unaffected.

### Interactive mHealth Apps for Patients Equipped With or Without the Chatbot Function

This study tested the usability of the I-C-MAUQ on the *Left-handed Doctor* app, which is empowered with the chatbot function. By contrast, Zhao et al [14] adopted the *Good Doctor* app, which was not equipped with the chatbot function. This difference in apps may somewhat explain the notable discrepancy in the questionnaire’s internal consistency and reliability between this study (Cronbach α=.988) and that by Zhao et al [14] (Cronbach α=.912). The mHealth apps used in Krebs and Duncan [7] and Mustafa et al [13] did not have the chatbot function. Thus, further research needs to be conducted to pinpoint the impact of this function on the usability of mHealth apps.

### Implications

It is worth adapting established and appropriate questionnaires with recorded validity because designing a new one is effort-, time-, and cost-consuming [12]. Proper translation and adaptation and TA [16,28] are essential to ensure equivalence between the original questionnaire and the translated version. Cultural and linguistic sensitivity is a prerequisite for ironing out the translation problems resulting from cultural and linguistic differences and making the translated questionnaire culturally relevant and appropriate. Therefore, qualified translators highly proficient in the source and target languages and health educators or practitioners need to make joint efforts to complete this challenging task.

Validation is crucial for ensuring the equivalence between the original version and the translated one. Content validity index has been used to quantify the questionnaire validity in some studies [9,13,15,29,30]. It has been widely used because of its simple measurement, accessibility, power to provide details for each item, and indication of item modification or deletion [30].

### Limitations

This study has several limitations. First, the convenient sampling of college students from a single university made it challenging to generalize the findings to the whole population in China. The recruitment of only healthy students also made the generalization of the results less convincing. Finally, the sample size was not sufficiently large to guarantee the generalization of findings.

### Conclusions

The I-C-MAUQ is highly reliable and valid for the *Left-handed Doctor* app, and thus suitable for testing the usability of interactive mHealth apps used by patients in China. This finding is in line with the study by Marzuki et al [12], further confirming the cross-cultural validity, reliability, and adaptability of the MAUQ. We identified certain factors that influence the perceived usability of mHealth apps, including users’ age, gender, education, profession, and possibly previous experience with mHealth apps as well as the chatbot function of such apps. Most notably, we found a wider acceptance of this new technology among young Chinese female college students who were more engaged in the interaction with health care chatbots. The age-, gender- and profession-induced preference for new digital health interventions in China aligns with the findings from other similar studies in the United States [9] and Malaysia [13]. This preference identifies areas for further research on the social, cultural, and gender adaptation of health technologies.

## Appendix

Multimedia Appendix 1 I-C-MAUQ, together with the C-MAUQ and the MAUQ. See also [31].

Multimedia Appendix 2 Informants' demographics.

Multimedia Appendix 3 Additional tables.

Multimedia Appendix 4 Frequency analysis of the usability of the *Left-handed Doctor* app.

## Abbreviations

CCA: cross-cultural adaptation
CITC: the corrected item-total correlation
C-MAUQ: the Chinese version of the MAUQ
CR: critical value
I-C-MAUQ: the improved C-MAUQ
MAUQ: mHealth App Usability Questionnaire
mHealth: mobile health
PSSUQ: Post-Study System Usability Questionnaire
RI: random index
SUS: System Usability Scale
TA: translatability assessment
