# Supplementary material for: Chinese Version of the Mobile Health App Usability Questionnaire: Translation, Adaptation, and Validation Study
Source: JMIR Form Res. 2022 Jul 6;6(7):e37933. doi: 10.2196/37933 (PMC9301561; doi:10.2196/37933)
Supplement: Multimedia Appendix 1 [file formative_v6i7e37933_app1.docx]

**Multimedia Appendix 1. I-C-MAUQ, together with the C-MAUQ and the MAUQ**

**The MAUQ**

mHealth App Usability Questionnaire (MAUQ) for Interactive mHealth Apps Used by Patients

| statements | 1 | 2 | 3 | 4 | 5 | 6 | 7 |
| --- | --- | --- | --- | --- | --- | --- | --- |
| 1. The app was easy to use |  |  |  |  |  |  |  |
| 2. It was easy for me to learn to use the app |  |  |  |  |  |  |  |
| 3. I like the interface of the app |  |  |  |  |  |  |  |
| 4.The information in the app was well organized, so I could easily find the information I needed |  |  |  |  |  |  |  |
| 5. I feel comfortable using this app in social settings. |  |  |  |  |  |  |  |
| 6. The amount of time involved in using this app has been fitting for me |  |  |  |  |  |  |  |
| 7. I would use this app again |  |  |  |  |  |  |  |
| 8. Overall, I am satisfied with this app |  |  |  |  |  |  |  |
| 9. Whenever I made a mistake using the app, I could recover easily and quickly |  |  |  |  |  |  |  |
| 10. This mHealth app provided an acceptable way to receive health care services |  |  |  |  |  |  |  |
| 11. The app adequately acknowledged and provided information to let me know the progress of my action |  |  |  |  |  |  |  |
| 12. The navigation was consistent when moving between screens |  |  |  |  |  |  |  |
| 13. The interface of the app allowed me to use all the functions (such as entering information, responding to reminders, viewing information) offered by the app |  |  |  |  |  |  |  |
| 14. This app has all the functions and capabilities I expected it to have |  |  |  |  |  |  |  |
| 15. The app would be useful for my health and well-being |  |  |  |  |  |  |  |
| 16. The app improved my access to healthcare services |  |  |  |  |  |  |  |
| 17. The app helped me manage my health effectively |  |  |  |  |  |  |  |
| 18. The app made it convenient for me to communicate with my healthcare provider |  |  |  |  |  |  |  |
| 19. Using the app, I had many more opportunities to interact with my healthcare provider |  |  |  |  |  |  |  |
| 20. I felt confident that any information I sent to my provider using the app would be received |  |  |  |  |  |  |  |
| 21. I felt comfortable communicating with my healthcare provider using the app |  |  |  |  |  |  |  |

Supplementary ：In this questionnaire, 1 - strongly agree, 2 –agree, 3 – somewhat agree, 4 – neither agree nor disagree, 5 – somewhat disagree, 6 – disagree, 7 – strongly disagree

To determine the usability of an app, calculate the total and determine the average of the responses to all statements. The closer the average to 1, the higher the usability of the app.

**The C-MAUQ**

| 条目 | 1 | 2 | 3 | 4 | 5 | 6 | 7 |
| --- | --- | --- | --- | --- | --- | --- | --- |
| 1. 该应用程序是容易使用的 |  |  |  |  |  |  |  |
| 2. 对我来说，学习使用该应用程序是容易的 |  |  |  |  |  |  |  |
| 3. 我喜欢该应用程序的界面 |  |  |  |  |  |  |  |
| 4. 该应用程序里的信息非常具有条理性，我可以很容易找到我需要的信息 |  |  |  |  |  |  |  |
| 5. 我在各种公共场合都能自在的使用该应用程序 |  |  |  |  |  |  |  |
| 6. 使用该应用程序不会占用我太多时间 |  |  |  |  |  |  |  |
| 7. 我会再次使用该应用程序 |  |  |  |  |  |  |  |
| 8. 总体来说，我对该应用程序是满意的 |  |  |  |  |  |  |  |
| 9. 每当我在使用该应用程序有一个错误操作时，我可以轻易并且很快的修正过来 |  |  |  |  |  |  |  |
| 10. 这款移动医疗应用提供医疗保健服务的方式易于让用户接受 |  |  |  |  |  |  |  |
| 11. 该应用程序给出足够的反馈和信息，以便让我知道我的步骤所处的进度 |  |  |  |  |  |  |  |
| 12. 页面切换的方式和过程是统一的 |  |  |  |  |  |  |  |
| 13. 该应用程序的界面允许我使用它提供的所有功能(比如输入信息，回应提醒，和阅读信息） |  |  |  |  |  |  |  |
| 14. 该应用程序拥有我所期待的所有功能和处理能力 |  |  |  |  |  |  |  |
| 15. 该应用程序对我的健康有益 |  |  |  |  |  |  |  |
| 16. 该应用程序改善了我获得医疗服务的途径 |  |  |  |  |  |  |  |
| 17. 该应用程序有助于我更有效的管理我的健康状况 |  |  |  |  |  |  |  |
| 18. 该应用程序使我与我的医护人员的交流变得方便 |  |  |  |  |  |  |  |
| 19. 使用该应用程序，让我有了更多和我的医护人员互相交流的机会 |  |  |  |  |  |  |  |
| 20. 我相信我的医护人员能收到我使用该应用程序发送的任何信息 |  |  |  |  |  |  |  |
| 21. 我感觉使用该应用程序与我的医护人员交流很舒服 |  |  |  |  |  |  |  |

Chinese version of mHealth App Usability Questionnaire (MAUQ) for Interactive mHealth Apps Used by Patients

补充：在本问卷中，1-非常同意，2-同意，3-有些同意，4-一般，5-有些不同意，6-不同意，7-非常不同意

要确定应用程序的可用性，请计算所有条目的相应总数并确定其平均值。平均值越接近1，应用程序的可用性就越高。

**The I-C-MAUQ**

Chinese version of mHealth App Usability Questionnaire (MAUQ) for Interactive mHealth Apps Used by Patients. Blue indicates modifications.

| 条目 | 1 | 2 | 3 | 4 | 5 | 6 | 7 |
| --- | --- | --- | --- | --- | --- | --- | --- |
| 1. 这个应用程序容易使用。 |  |  |  |  |  |  |  |
| 2. 我觉得学习使用该应用程序很容易。 |  |  |  |  |  |  |  |
| 3. 我喜欢该应用程序的界面。 |  |  |  |  |  |  |  |
| 4. 该应用程序里的信息非常具有条理性，我可以很容易找到我需要的信息。 |  |  |  |  |  |  |  |
| 5. 我在各种公共场合都能随意使用该应用程序。 |  |  |  |  |  |  |  |
| 6. 使用该应用程序不会占用我太多时间。 |  |  |  |  |  |  |  |
| 7. 我会再次使用该应用程序。 |  |  |  |  |  |  |  |
| 8. 总体来说，我对该应用程序是满意的。 |  |  |  |  |  |  |  |
| 9. 在使用该程序过程中，当我操作不当或操作错误时，我可以很容地马上调整过来。 |  |  |  |  |  |  |  |
| 10. 这款移动医疗应用提供医疗保健服务的方式易于让用户接受。 |  |  |  |  |  |  |  |
| 11. 该应用程序能提供足够的反馈和信息，便于我了解每一步操作程序与进度。 |  |  |  |  |  |  |  |
| 12. 该程序页面切换的方式与过程是统一的。 |  |  |  |  |  |  |  |
| 13. 该应用程序允许我使用界面上提供的所有功能(比如，信息输入，回应提醒，信息浏览，等等）。 |  |  |  |  |  |  |  |
| 14. 该应用程序拥有我所期待的所有功能与应对能力。 |  |  |  |  |  |  |  |
| 15. 该应用程序对我的健康有益。 |  |  |  |  |  |  |  |
| 16. 该应用程序改善了我获得医疗服务的途径。 |  |  |  |  |  |  |  |
| 17. 该应用程序有助于我更有效地管理我的健康状况。 |  |  |  |  |  |  |  |
| 18. 该应用程序方便我与医护人员交流。 |  |  |  |  |  |  |  |
| 19. 使用该应用程序，让我有了更多与医护人员交流的机会。 |  |  |  |  |  |  |  |
| 20. 我相信医护人员能收到我使用该应用程序发送的任何信息。 |  |  |  |  |  |  |  |
| 21. 我觉得使用该应用程序与医护人员交流很轻松愉快。 |  |  |  |  |  |  |  |

补充：在本问卷中，1-非常同意，2-同意，3-有些同意，4-一般，5-有些不同意，6-不同意，7-非常不同意

要确定应用程序的可用性，请计算所有条目的相应总数并确定其平均值。平均值越接近1，应用程序的可用性就越高。

Hao K. The pandemic is emptying call centers. AI chatbots are swooping in. MIT Technology Review. 2022. https://www.technologyreview.com/2020/05/14/1001716/ai-chatbots-take-call-center-jobs-during-coronaviruspandemic.
