# Supplementary material for: Chinese Version of the Mobile Health App Usability Questionnaire: Translation, Adaptation, and Validation Study
Source: JMIR Form Res. 2022 Jul 6;6(7):e37933. doi: 10.2196/37933 (PMC9301561; doi:10.2196/37933)
Supplement: Multimedia Appendix 2 [file formative_v6i7e37933_app2.docx]

| **Description of Demographics** | | | | |
| --- | --- | --- | --- | --- |
| **Items** | **Categories** | **N of Sample** | **Percent (%)** | **Cumulative Percent (%)** |
| I’m a____. | Freshman | 64 | 19.88 | 19.88 |
|  | Sophomore | 29 | 9.01 | 28.88 |
|  | Junior | 88 | 27.33 | 56.21 |
|  | Senior | 48 | 14.91 | 71.12 |
|  | Graduate.One ^✷^ | 46 | 14.29 | 85.40 |
|  | Graduate.Two ^✷^ | 47 | 14.60 | 100.00 |
| I’m ____ years old. | 18 | 13 | 4.04 | 4.04 |
|  | 19 | 47 | 14.60 | 18.63 |
|  | 20 | 42 | 13.04 | 31.68 |
|  | 21 | 60 | 18.63 | 50.31 |
|  | 22 | 60 | 18.63 | 68.94 |
|  | 23 | 42 | 13.04 | 81.99 |
|  | 24 | 23 | 7.14 | 89.13 |
|  | 25 | 16 | 4.97 | 94.10 |
|  | 26 | 13 | 4.04 | 98.14 |
|  | 27 | 1 | 0.31 | 98.45 |
|  | 28 | 1 | 0.31 | 98.76 |
|  | 29 | 1 | 0.31 | 99.07 |
|  | 30 | 1 | 0.31 | 99.38 |
|  | 32 | 1 | 0.31 | 99.69 |
|  | 33 | 1 | 0.31 | 100.00 |
| I’m ____ . | male | 30 | 9.32 | 9.32 |
|  | female | 292 | 90.68 | 100.00 |
| Facing the COVID-19, I mainly obtain health care information through____. | visiting a doctor | 138 | 42.86 | 42.86 |
|  | logging onto the Internet | 114 | 35.40 | 78.26 |
|  | reading books, papers, and journals | 7 | 2.17 | 80.43 |
|  | families, friends, and classmates | 54 | 16.77 | 97.20 |
|  | mHealth apps | 9 | 2.80 | 100.00 |
| Total | | 322 | 100.0 | 100.0 |

^✷^Graduate.One and Graduate.Two stand for the first-year and second-year postgraduate students respectively.
