# Supplementary material for: Chinese Version of the Mobile Health App Usability Questionnaire: Translation, Adaptation, and Validation Study
Source: JMIR Form Res. 2022 Jul 6;6(7):e37933. doi: 10.2196/37933 (PMC9301561; doi:10.2196/37933)
Supplement: Multimedia Appendix 3 [file formative_v6i7e37933_app3.docx]

**Multimedia Appendix 3.** Additional tables.

**Table S1.** Pearson correlation between the 21 items in the questionnaire.

| Mean (SD) | Items | 1 | 2 | 3 | 4 | 5 | 6 | 7 | 8 | 9 | 10 | 11 | 12 | 13 | 14 | 15 | 16 | 17 | 18 | 19 | 20 | 21 |
| --- | --- | --- | --- | --- | --- | --- | --- | --- | --- | --- | --- | --- | --- | --- | --- | --- | --- | --- | --- | --- | --- | --- |
| 2.373 (1.18) | 1 | 1 |  |  |  |  |  |  |  |  |  |  |  |  |  |  |  |  |  |  |  |  |
| 2.224 (1.079) | 2 | 0.832^a^ | 1 |  |  |  |  |  |  |  |  |  |  |  |  |  |  |  |  |  |  |  |
| 2.307 (1.125) | 3 | 0.849^a^ | 0.836^a^ | 1 |  |  |  |  |  |  |  |  |  |  |  |  |  |  |  |  |  |  |
| 2.354 (1.16) | 4 | 0.861^a^ | 0.805^a^ | 0.862^a^ | 1 |  |  |  |  |  |  |  |  |  |  |  |  |  |  |  |  |  |
| 2.379 (1.176) | 5 | 0.751^a^ | 0.770^a^ | 0.799^a^ | 0.811^a^ | 1 |  |  |  |  |  |  |  |  |  |  |  |  |  |  |  |  |
| 2.348 (1.17) | 6 | 0.784^a^ | 0.807^a^ | 0.797^a^ | 0.782^a^ | 0.783^a^ | 1 |  |  |  |  |  |  |  |  |  |  |  |  |  |  |  |
| 2.497 (1.246) | 7 | 0.784^a^ | 0.758^a^ | 0.808^a^ | 0.803^a^ | 0.768^a^ | 0.798^a^ | 1 |  |  |  |  |  |  |  |  |  |  |  |  |  |  |
| 2.307 (1.136) | 8 | 0.820^a^ | 0.787^a^ | 0.825^a^ | 0.875^a^ | 0.819^a^ | 0.819^a^ | 0.860^a^ | 1 |  |  |  |  |  |  |  |  |  |  |  |  |  |
| 2.366 (1.182) | 9 | 0.768^a^ | 0.802^a^ | 0.802^a^ | 0.805^a^ | 0.776^a^ | 0.804^a^ | 0.785^a^ | 0.802^a^ | 1 |  |  |  |  |  |  |  |  |  |  |  |  |
| 2.314 (1.14) | 10 | 0.816^a^ | 0.811^a^ | 0.847^a^ | 0.849^a^ | 0.796^a^ | 0.815^a^ | 0.855^a^ | 0.868^a^ | 0.818^a^ | 1 |  |  |  |  |  |  |  |  |  |  |  |
| 2.230 (1.101) | 11 | 0.819^a^ | 0.853^a^ | 0.866^a^ | 0.832^a^ | 0.773^a^ | 0.840^a^ | 0.827^a^ | 0.857^a^ | 0.830^a^ | 0.925^a^ | 1 |  |  |  |  |  |  |  |  |  |  |
| 2.286 (1.07) | 12 | 0.806^a^ | 0.808^a^ | 0.804^a^ | 0.837^a^ | 0.797^a^ | 0.799^a^ | 0.792^a^ | 0.860^a^ | 0.786^a^ | 0.843^a^ | 0.859^a^ | 1 |  |  |  |  |  |  |  |  |  |
| 2.230 (1.089) | 13 | 0.779^a^ | 0.796^a^ | 0.793^a^ | 0.826^a^ | 0.807^a^ | 0.785^a^ | 0.774^a^ | 0.839^a^ | 0.762^a^ | 0.820^a^ | 0.842^a^ | 0.924^a^ | 1 |  |  |  |  |  |  |  |  |
| 2.366 (1.153) | 14 | 0.760^a^ | 0.798^a^ | 0.820^a^ | 0.788^a^ | 0.750^a^ | 0.795^a^ | 0.827^a^ | 0.820^a^ | 0.797^a^ | 0.851^a^ | 0.886^a^ | 0.834^a^ | 0.823^a^ | 1 |  |  |  |  |  |  |  |
| 2.317 (1.132) | 15 | 0.732^a^ | 0.780^a^ | 0.789^a^ | 0.792^a^ | 0.735^a^ | 0.782^a^ | 0.787^a^ | 0.820^a^ | 0.774^a^ | 0.796^a^ | 0.854^a^ | 0.832^a^ | 0.814^a^ | 0.879^a^ | 1 |  |  |  |  |  |  |
| 2.292 (1.153) | 16 | 0.700^a^ | 0.739^a^ | 0.742^a^ | 0.752^a^ | 0.720^a^ | 0.712^a^ | 0.734^a^ | 0.778^a^ | 0.721^a^ | 0.769^a^ | 0.809^a^ | 0.788^a^ | 0.792^a^ | 0.826^a^ | 0.869^a^ | 1 |  |  |  |  |  |
| 2.323 (1.117) | 17 | 0.735^a^ | 0.764^a^ | 0.776^a^ | 0.789^a^ | 0.732^a^ | 0.760^a^ | 0.775^a^ | 0.808^a^ | 0.743^a^ | 0.811^a^ | 0.847^a^ | 0.843^a^ | 0.825^a^ | 0.842^a^ | 0.902^a^ | 0.907^a^ | 1 |  |  |  |  |
| 2.342 (1.125) | 18 | 0.741^a^ | 0.776^a^ | 0.802^a^ | 0.755^a^ | 0.719^a^ | 0.771^a^ | 0.774^a^ | 0.780^a^ | 0.774^a^ | 0.817^a^ | 0.847^a^ | 0.816^a^ | 0.790^a^ | 0.861^a^ | 0.861^a^ | 0.840^a^ | 0.869^a^ | 1 |  |  |  |
| 2.320 (1.119) | 19 | 0.723^a^ | 0.766^a^ | 0.775^a^ | 0.748^a^ | 0.694^a^ | 0.750^a^ | 0.777^a^ | 0.765^a^ | 0.780^a^ | 0.785^a^ | 0.800^a^ | 0.805^a^ | 0.780^a^ | 0.855^a^ | 0.846^a^ | 0.823^a^ | 0.832^a^ | 0.920^a^ | 1 |  |  |
| 2.463 (1.166) | 20 | 0.694^a^ | 0.688^a^ | 0.711^a^ | 0.754^a^ | 0.695^a^ | 0.720^a^ | 0.761^a^ | 0.777^a^ | 0.727^a^ | 0.774^a^ | 0.769^a^ | 0.795^a^ | 0.755^a^ | 0.812^a^ | 0.819^a^ | 0.787^a^ | 0.806^a^ | 0.851^a^ | 0.853^a^ | 1 |  |
| 2.329 (1.137) | 21 | 0.753^a^ | 0.762^a^ | 0.741^a^ | 0.819^a^ | 0.759^a^ | 0.776^a^ | 0.755^a^ | 0.823^a^ | 0.746^a^ | 0.816^a^ | 0.800^a^ | 0.831^a^ | 0.819^a^ | 0.818^a^ | 0.828^a^ | 0.813^a^ | 0.846^a^ | 0.876^a^ | 0.857^a^ | 0.870^a^ | 1 |

^a^*P*<.01.

**Table S2.** Judgment matrix of the 21 items in the questionnaire.

| Mean | Items | 1 | 2 | 3 | 4 | 5 | 6 | 7 | 8 | 9 | 10 | 11 | 12 | 13 | 14 | 15 | 16 | 17 | 18 | 19 | 20 | 21 |
| --- | --- | --- | --- | --- | --- | --- | --- | --- | --- | --- | --- | --- | --- | --- | --- | --- | --- | --- | --- | --- | --- | --- |
| 2.373 | 1 | 1 | 1.067 | 1.028 | 1.008 | 0.997 | 1.011 | 0.950 | 1.028 | 1.003 | 1.026 | 1.064 | 1.038 | 1.064 | 1.003 | 1.024 | 1.035 | 1.021 | 1.013 | 1.023 | 0.963 | 1.019 |
| 2.224 | 2 | 0.937 | 1 | 0.964 | 0.945 | 0.935 | 0.947 | 0.891 | 0.964 | 0.940 | 0.961 | 0.997 | 0.973 | 0.997 | 0.940 | 0.960 | 0.970 | 0.957 | 0.950 | 0.959 | 0.903 | 0.955 |
| 2.307 | 3 | 0.973 | 1.038 | 1 | 0.980 | 0.970 | 0.983 | 0.924 | 1 | 0.975 | 0.997 | 1.035 | 1.010 | 1.035 | 0.975 | 0.996 | 1.007 | 0.993 | 0.985 | 0.995 | 0.937 | 0.991 |
| 2.354 | 4 | 0.992 | 1.059 | 1.020 | 1 | 0.990 | 1.003 | 0.943 | 1.020 | 0.995 | 1.017 | 1.056 | 1.030 | 1.056 | 0.995 | 1.016 | 1.027 | 1.013 | 1.005 | 1.015 | 0.956 | 1.011 |
| 2.379 | 5 | 1.003 | 1.070 | 1.031 | 1.011 | 1 | 1.013 | 0.953 | 1.031 | 1.005 | 1.028 | 1.067 | 1.041 | 1.067 | 1.005 | 1.027 | 1.038 | 1.024 | 1.016 | 1.025 | 0.966 | 1.021 |
| 2.348 | 6 | 0.990 | 1.056 | 1.017 | 0.997 | 0.987 | 1 | 0.940 | 1.017 | 0.992 | 1.015 | 1.053 | 1.027 | 1.053 | 0.992 | 1.013 | 1.024 | 1.011 | 1.003 | 1.012 | 0.953 | 1.008 |
| 2.497 | 7 | 1.052 | 1.123 | 1.082 | 1.061 | 1.050 | 1.063 | 1 | 1.082 | 1.055 | 1.079 | 1.120 | 1.092 | 1.120 | 1.055 | 1.078 | 1.089 | 1.075 | 1.066 | 1.076 | 1.014 | 1.072 |
| 2.307 | 8 | 0.973 | 1.038 | 1 | 0.980 | 0.970 | 0.983 | 0.924 | 1 | 0.975 | 0.997 | 1.035 | 1.010 | 1.035 | 0.975 | 0.996 | 1.007 | 0.993 | 0.985 | 0.995 | 0.937 | 0.991 |
| 2.366 | 9 | 0.997 | 1.064 | 1.026 | 1.005 | 0.995 | 1.008 | 0.948 | 1.026 | 1 | 1.023 | 1.061 | 1.035 | 1.061 | 1 | 1.021 | 1.033 | 1.019 | 1.011 | 1.020 | 0.961 | 1.016 |
| 2.314 | 10 | 0.975 | 1.041 | 1.003 | 0.983 | 0.973 | 0.985 | 0.927 | 1.003 | 0.978 | 1 | 1.038 | 1.012 | 1.038 | 0.978 | 0.999 | 1.009 | 0.996 | 0.988 | 0.997 | 0.939 | 0.993 |
| 2.230 | 11 | 0.940 | 1.003 | 0.966 | 0.947 | 0.937 | 0.950 | 0.893 | 0.966 | 0.942 | 0.964 | 1 | 0.976 | 1 | 0.942 | 0.962 | 0.973 | 0.960 | 0.952 | 0.961 | 0.905 | 0.957 |
| 2.286 | 12 | 0.963 | 1.028 | 0.991 | 0.971 | 0.961 | 0.974 | 0.915 | 0.991 | 0.966 | 0.988 | 1.025 | 1 | 1.025 | 0.966 | 0.987 | 0.997 | 0.984 | 0.976 | 0.985 | 0.928 | 0.981 |
| 2.230 | 13 | 0.940 | 1.003 | 0.966 | 0.947 | 0.937 | 0.950 | 0.893 | 0.966 | 0.942 | 0.964 | 1 | 0.976 | 1 | 0.942 | 0.962 | 0.973 | 0.960 | 0.952 | 0.961 | 0.905 | 0.957 |
| 2.366 | 14 | 0.997 | 1.064 | 1.026 | 1.005 | 0.995 | 1.008 | 0.948 | 1.026 | 1 | 1.023 | 1.061 | 1.035 | 1.061 | 1 | 1.021 | 1.033 | 1.019 | 1.011 | 1.020 | 0.961 | 1.016 |
| 2.317 | 15 | 0.976 | 1.042 | 1.004 | 0.984 | 0.974 | 0.987 | 0.928 | 1.004 | 0.979 | 1.001 | 1.039 | 1.014 | 1.039 | 0.979 | 1 | 1.011 | 0.997 | 0.989 | 0.999 | 0.941 | 0.995 |
| 2.292 | 16 | 0.966 | 1.031 | 0.993 | 0.974 | 0.963 | 0.976 | 0.918 | 0.993 | 0.969 | 0.991 | 1.028 | 1.003 | 1.028 | 0.969 | 0.989 | 1 | 0.987 | 0.979 | 0.988 | 0.931 | 0.984 |
| 2.323 | 17 | 0.979 | 1.045 | 1.007 | 0.987 | 0.977 | 0.989 | 0.930 | 1.007 | 0.982 | 1.004 | 1.042 | 1.016 | 1.042 | 0.982 | 1.003 | 1.014 | 1 | 0.992 | 1.001 | 0.943 | 0.997 |
| 2.342 | 18 | 0.987 | 1.053 | 1.015 | 0.995 | 0.984 | 0.997 | 0.938 | 1.015 | 0.990 | 1.012 | 1.050 | 1.024 | 1.050 | 0.990 | 1.011 | 1.022 | 1.008 | 1 | 1.009 | 0.951 | 1.005 |
| 2.320 | 19 | 0.978 | 1.043 | 1.005 | 0.985 | 0.975 | 0.988 | 0.929 | 1.005 | 0.980 | 1.003 | 1.040 | 1.015 | 1.040 | 0.980 | 1.001 | 1.012 | 0.999 | 0.991 | 1 | 0.942 | 0.996 |
| 2.463 | 20 | 1.038 | 1.108 | 1.067 | 1.046 | 1.035 | 1.049 | 0.986 | 1.067 | 1.041 | 1.064 | 1.104 | 1.077 | 1.104 | 1.041 | 1.063 | 1.075 | 1.060 | 1.052 | 1.062 | 1 | 1.057 |
| 2.329 | 21 | 0.982 | 1.047 | 1.009 | 0.989 | 0.979 | 0.992 | 0.933 | 1.009 | 0.984 | 1.007 | 1.045 | 1.019 | 1.045 | 0.984 | 1.005 | 1.016 | 1.003 | 0.995 | 1.004 | 0.946 | 1 |
