# Supplementary material for: Chinese Version of the Mobile Health App Usability Questionnaire: Translation, Adaptation, and Validation Study
Source: JMIR Form Res. 2022 Jul 6;6(7):e37933. doi: 10.2196/37933 (PMC9301561; doi:10.2196/37933)
Supplement: Multimedia Appendix 4 [file formative_v6i7e37933_app4.docx]

| **Frequency** | | | | |
| --- | --- | --- | --- | --- |
| **Items** | **Categories** | **N of Samples** | **Percent (%)** | **Cumulative Percent (%)** |
| 1 | SA ^✷^ | 73 | 22.67 | 22.67 |
|  | A ^✷^ | 132 | 40.99 | 63.66 |
|  | SWA ^✷^ | 62 | 19.25 | 82.92 |
|  | N ^✷^ | 46 | 14.29 | 97.20 |
|  | SWD ^✷^ | 1 | 0.31 | 97.52 |
|  | D ^✷^ | 4 | 1.24 | 98.76 |
|  | SD ^✷^ | 4 | 1.24 | 100.00 |
| 2 | SA | 79 | 24.53 | 24.53 |
|  | A | 144 | 44.72 | 69.25 |
|  | SWA | 62 | 19.25 | 88.51 |
|  | N | 31 | 9.63 | 98.14 |
|  | SWD | 1 | 0.31 | 98.45 |
|  | D | 1 | 0.31 | 98.76 |
|  | SD | 4 | 1.24 | 100.00 |
| 3 | SA | 76 | 23.60 | 23.60 |
|  | A | 133 | 41.30 | 64.91 |
|  | SWA | 68 | 21.12 | 86.02 |
|  | N | 38 | 11.80 | 97.83 |
|  | SWD | 1 | 0.31 | 98.14 |
|  | D | 2 | 0.62 | 98.76 |
|  | SD | 4 | 1.24 | 100.00 |
| 4 | SA | 70 | 21.74 | 21.74 |
|  | A | 136 | 42.24 | 63.98 |
|  | SWA | 71 | 22.05 | 86.02 |
|  | N | 35 | 10.87 | 96.89 |
|  | SWD | 2 | 0.62 | 97.52 |
|  | D | 3 | 0.93 | 98.45 |
|  | SD | 5 | 1.55 | 100.00 |
| 5 | SA | 74 | 22.98 | 22.98 |
|  | A | 125 | 38.82 | 61.80 |
|  | SWA | 72 | 22.36 | 84.16 |
|  | N | 40 | 12.42 | 96.58 |
|  | SWD | 4 | 1.24 | 97.83 |
|  | D | 3 | 0.93 | 98.76 |
|  | SD | 4 | 1.24 | 100.00 |
| 6 | SA | 73 | 22.67 | 22.67 |
|  | A | 133 | 41.30 | 63.98 |
|  | SWA | 71 | 22.05 | 86.02 |
|  | N | 33 | 10.25 | 96.27 |
|  | SWD | 5 | 1.55 | 97.83 |
|  | D | 2 | 0.62 | 98.45 |
|  | SD | 5 | 1.55 | 100.00 |
| 7 | SA | 70 | 21.74 | 21.74 |
|  | A | 113 | 35.09 | 56.83 |
|  | SWA | 76 | 23.60 | 80.43 |
|  | N | 50 | 15.53 | 95.96 |
|  | SWD | 3 | 0.93 | 96.89 |
|  | D | 5 | 1.55 | 98.45 |
|  | SD | 5 | 1.55 | 100.00 |
| 8 | SA | 74 | 22.98 | 22.98 |
|  | A | 136 | 42.24 | 65.22 |
|  | SWA | 72 | 22.36 | 87.58 |
|  | N | 31 | 9.63 | 97.20 |
|  | SWD | 2 | 0.62 | 97.83 |
|  | D | 2 | 0.62 | 98.45 |
|  | SD | 5 | 1.55 | 100.00 |
| 9 | SA | 75 | 23.29 | 23.29 |
|  | A | 128 | 39.75 | 63.04 |
|  | SWA | 67 | 20.81 | 83.85 |
|  | N | 41 | 12.73 | 96.58 |
|  | SWD | 5 | 1.55 | 98.14 |
|  | D | 1 | 0.31 | 98.45 |
|  | SD | 5 | 1.55 | 100.00 |
| 10 | SA | 76 | 23.60 | 23.60 |
|  | A | 132 | 40.99 | 64.60 |
|  | SWA | 71 | 22.05 | 86.65 |
|  | N | 34 | 10.56 | 97.20 |
|  | SWD | 2 | 0.62 | 97.83 |
|  | D | 3 | 0.93 | 98.76 |
|  | SD | 4 | 1.24 | 100.00 |
| 11 | SA | 81 | 25.16 | 25.16 |
|  | A | 138 | 42.86 | 68.01 |
|  | SWA | 69 | 21.43 | 89.44 |
|  | SWD | 27 | 8.39 | 97.83 |
|  | D | 3 | 0.93 | 98.76 |
|  | SD | 4 | 1.24 | 100.00 |
| 12 | SA | 71 | 22.05 | 22.05 |
|  | A | 140 | 43.48 | 65.53 |
|  | SWA | 74 | 22.98 | 88.51 |
|  | N | 31 | 9.63 | 98.14 |
|  | SWD | 1 | 0.31 | 98.45 |
|  | D | 1 | 0.31 | 98.76 |
|  | SD | 4 | 1.24 | 100.00 |
| 13 | SA | 81 | 25.16 | 25.16 |
|  | A | 137 | 42.55 | 67.70 |
|  | SWA | 70 | 21.74 | 89.44 |
|  | N | 26 | 8.07 | 97.52 |
|  | SWD | 3 | 0.93 | 98.45 |
|  | D | 1 | 0.31 | 98.76 |
|  | SD | 4 | 1.24 | 100.00 |
| 14 | SA | 73 | 22.67 | 22.67 |
|  | A | 125 | 38.82 | 61.49 |
|  | SWA | 79 | 24.53 | 86.02 |
|  | N | 33 | 10.25 | 96.27 |
|  | SWD | 6 | 1.86 | 98.14 |
|  | D | 2 | 0.62 | 98.76 |
|  | SD | 4 | 1.24 | 100.00 |
| 15 | SA | 74 | 22.98 | 22.98 |
|  | A | 134 | 41.61 | 64.60 |
|  | SWA | 72 | 22.36 | 86.96 |
|  | N | 33 | 10.25 | 97.20 |
|  | SWD | 2 | 0.62 | 97.83 |
|  | D | 3 | 0.93 | 98.76 |
|  | SD | 4 | 1.24 | 100.00 |
| 16 | SA | 77 | 23.91 | 23.91 |
|  | A | 139 | 43.17 | 67.08 |
|  | SWA | 64 | 19.88 | 86.96 |
|  | N | 30 | 9.32 | 96.27 |
|  | SWD | 5 | 1.55 | 97.83 |
|  | D | 3 | 0.93 | 98.76 |
|  | SD | 4 | 1.24 | 100.00 |
| 17 | SA | 71 | 22.05 | 22.05 |
|  | A | 136 | 42.24 | 64.29 |
|  | SWA | 75 | 23.29 | 87.58 |
|  | N | 30 | 9.32 | 96.89 |
|  | SWD | 4 | 1.24 | 98.14 |
|  | D | 2 | 0.62 | 98.76 |
|  | SD | 4 | 1.24 | 100.00 |
| 18 | SA | 76 | 23.60 | 23.60 |
|  | A | 122 | 37.89 | 61.49 |
|  | SWA | 78 | 24.22 | 85.71 |
|  | N | 39 | 12.11 | 97.83 |
|  | SWD | 2 | 0.62 | 98.45 |
|  | D | 1 | 0.31 | 98.76 |
|  | SD | 4 | 1.24 | 100.00 |
| 19 | SA | 77 | 23.91 | 23.91 |
|  | A | 126 | 39.13 | 63.04 |
|  | SWA | 73 | 22.67 | 85.71 |
|  | N | 40 | 12.42 | 98.14 |
|  | SWD | 1 | 0.31 | 98.45 |
|  | D | 1 | 0.31 | 98.76 |
|  | SD | 4 | 1.24 | 100.00 |
| 20 | SA | 67 | 20.81 | 20.81 |
|  | A | 120 | 37.27 | 58.07 |
|  | SWA | 71 | 22.05 | 80.12 |
|  | N | 56 | 17.39 | 97.52 |
|  | SWD | 3 | 0.93 | 98.45 |
|  | D | 1 | 0.31 | 98.76 |
|  | SD | 4 | 1.24 | 100.00 |
| 21 | SA | 73 | 22.67 | 22.67 |
|  | A | 135 | 41.93 | 64.60 |
|  | SWA | 69 | 21.43 | 86.02 |
|  | N | 35 | 10.87 | 96.89 |
|  | SWD | 4 | 1.24 | 98.14 |
|  | D | 2 | 0.62 | 98.76 |
|  | SD | 4 | 1.24 | 100.00 |
| Total | | 322 | 100.0 | 100.0 |

^✷^SA, A, SWA, N, SWD, D, and SD stand for strongly agree, agree, somewhat agree, neither agree nor disagree, somewhat disagree, disagree, and strongly disagree, respectively.
